# Hepatic echinococcosis: A review

**DOI:** 10.1016/j.amsu.2018.10.032

**Published:** 2018-11-02

**Authors:** Namita Bhutani, Pradeep Kajal

**Affiliations:** a Dept. of Pathology, PGIMS Rohtak, Haryana, India; b Dept. of Paediatric Surgery, PGIMS Rohtak, Haryana, India

**Keywords:** Cystic echinococcosis, Epidemiology, Polycystic echinococcosis, Zoonoses

## Abstract

Cystic echinococcosis (CE) is a widely endemic helminthic disease caused by infection with metacestodes (larval stage) of the Echinococcus granulosus tapeworm and transmitted by dogs. *E. granulosus* are common parasites in certain parts of the world, and are present on every continent with the exception of Antarctica. As a result, a large number of people are affected by CE. In humans, the disease is characterized by slowly growing cyst commonly occurring in liver and lungs. Clinical features are mainly right upper quadrant pain, feeling of lump and enlarged tender liver. The cyst may be complicated by infection or rupture and may lead to anaphylactic reaction. The diagnosis depends on clinical suspicion. They appear in two ways as general (systemic) symptoms, and local symptoms based on the site and organ on which the larva settles. While cysts sometimes recover spontaneously, more severe clinical presentations are observed in immunosuppressed individuals. Ultrasonography supported by serology is the main diagnostic modality. The treatment varies from surgical intervention to minimally invasive treatments (percutaneous drainage) or medical therapies. Surgery is still the best treatment modality. Percutaneous drainage of the cyst is a good option in selected cases. New sensitive and specific diagnostic methods and effective therapeutic approaches against echinococcosis have been developed in the last 10 years.

## Highlights

• Echinococcal cysts, although fairly uncommon, should be considered in the differential diagnosis of hepatic cysts.
• Serum antibody assays generally have low sensitivities, but antigen assays may be of value.
• Imaging is crucial in determining cyst stage, size, location and complications.
• Uncomplicated active cysts can be managed with chemotherapy alone or in combination with a PAIR approach. Inactive cysts can be managed with the “watch-and-wait” strategy.
• Surgery is the first choice when there is cystobiliary fistula and significant extra-hepatic extension with high risk of perforation.

## 1 Introduction

Human echinococcosis is a zoonotic infection transmitted by dogs in livestock raising areas. The causative agent is Echinococcus granulosus belonging to taeniidae family of cestoda class [1]. Based on their genetic structures and biological properties, six different types of E. granulosis (sheep, cattle, horse, camel, swine and deer) have been shown, but four are of public health concern: *E. granulosus* (which causes Cystic Echinococcosis), E. multilocularis (which causes alveolar echinococcosis), E. vogeli and E. oligarthrus (which cause polycystic echinococcosis). Two new species have recently been identified: E. shiquicus in small mammals from the Tibetan plateau and E. felidis in African lions, but their zoonotic transmission potential is unknown. Molecular studies using mitochondrial DNA sequences have identified 10 distinct genetic types (G1—10) within *E. granulosus* [2,3]. These include two sheep strains (G1 and G2), two bovid strains (G3 and G5), a horse strain (G4), a camelid strain (G6), a pig strain (G7), and a cervid strain (G8). A ninth genotype (G9) has been described in swine in Poland and a tenth strain (G10) in reindeer in Eurasia. The sheep strain (G1) is the most cosmopolitan form and is most commonly associated with human infections [2]. Certain human activities (e.g. the widespread rural practice of feeding dogs the viscera of home-butchered sheep) facilitate transmission of the sheep strain and consequently raise the risk that humans will become infected [3]. (see Table 1)

**Table 1:** Imaging modalities used.

DIRECT X RAYS	Not diagnostic for hydatid cyst, but may lead to the suspicion of disease (Fig. 2). Complicated hydatid cyst: ‘air meniscus sign’ water-lily sign double-dome arch sign’ and ‘calcification’.
ULTRASONOGRAPHY	Gold standard diagnostic method screening method of choice post treatment monitoring highest sensitivity for the detection of membranes, septa, and hydatid sand
COMPUTED TOMOGRAPHY SCAN	Valuable information regarding the size of the cyst, septation presence, the integrity of germinative membrane, status of liver parenchyma, location and the depth of the cyst and adjacency with bile ducts (Fig. 1). The presence of daughter cysts and exogenous cysts can also clearly be seen on CT.
MAGNETIC RESONANCE IMAGING	Well structural details of the hydatid cysts, MRI can detect early irregularities in the wall, thought to represent an impending membrane detachment. MRI is especially indicated in cerebral pathologies.

There are two hosts in the life-cycle of the parasite. The first one is the “primary host” or definitive host, and the second one is the “intermediate host” in which the illness occurs. Adult forms are present in the intestines of primary hosts including cats, dogs, wolfs and foxes, and here, they only cause intestinal parasitosis but not organ disease. Adult parasite lives approximately for 5 months in dog intestines [4,5]. “Definitive hosts” spread millions of parasite eggs on defecation. Sheep and other herbivorous animals becomes “intermediate host” for the parasite when they eat herbs contaminated with these eggs, or humans become “intermediate host” for the parasite when they eat food contaminated with these eggs. Embryo (oncosphere) which comes out of the egg taken via gastrointestinal tract, adheres to intestinal wall with its hooks, then enters into circulation and reaches firstly to the liver. Thus, liver is the most common site of disease in humans accounting for 50–70% of cases, followed by the lungs (20–30%), and less frequently the spleen, kidneys, heart, bones, central nervous system, and other organs [5]. Embryo loses its scolex when it settles in an organ, and takes the cyst form consisting of cuticula (exocyst) and germinal membrane (endocyst). The cyst has sterile, clear fluid inside, and this cystic structure is wrapped with a fibrous capsule “pericyst”. When alive hydatid cysts are eaten by the last host dog, the infection chain is completed, and the life cycle returns to beginning [6,7].

Hepatic CE is life-threatening disease. Despite some progress in the control of echinococcosis, this zoonosis continues to be a major public health problem in several countries, and in several others it constitutes an emerging and re-emerging disease. In this review, we discuss aspects of the biology, life cycle, etiology, distribution, and transmission of the Echinococcus organisms, and the epidemiology, clinical features, treatment, and effect of improved diagnosis of the diseases they cause.

## 2 Epidemiology

According to the World Health Organization (WHO), *E. granulosus* is endemic in South America, Eastern Europe, Russia, the Middle East, and China, where human incidence rates are as high as 50 per 100,000 person-years. In certain areas, such as slaughter houses in South America, prevalence varies from 20% to as high as 95% [8]. The most common intermediate hosts are farm animals, such as sheep, goats, swine, camels, horses, and cattle, as well as mule deer [9]. The incidence of surgical cases reflects only a fraction of the number of infected hosts, which, in turn, is only a fraction of the actual prevalence in endemic areas. Foci of hydatid disease also exist in India where the highest prevalence is reported in Andhra Pradesh and Tamil Nadu than in other parts of the country [10,11]. Tanzania, Malta, South Cyprus and New Zealand became hydatid cyst free zones with their applied public health policies [12]. Factors such as agriculture-based subsistence, low socio-economic status, regional climate, and uncontrolled and unhygienic animal slaughtering increase the incidence.

## 3 Etiology and pathogenesis

Adult tapeworm lives in the upper small bowel of the definitive host (dogs). Other definitive hosts are wolves, jackals, domestic cats, and reindeer etc. Sheep, cattle, pigs and humans contain larval stage and are intermediate hosts. They are infected faeco-orally by eggs shed in the environment with faeces of infected dogs. Upon entering the small intestine, the parasite remains firmly attached to the mucosa, and later sheds gravid proglottids that are excreted in the infected animal's faeces [13]. Within each proglottid, there are hundreds of eggs. These eggs can then be ingested by intermediate hosts where they mature into cysts and daughter cysts, such as in sheep that acquire the infection by grazing upon grass contaminated with dog faeces containing the eggs. Human infection does not occur by the handling or ingestion of meat or viscera from infected sheep. Rather, humans are accidental intermediate hosts that become infected either by direct contact with a dog contaminated with egg-bearing faeces or by ingesting water, food, or soil contaminated with such faeces. In human infection, the first stage is the asymptomatic incubation period, during which ingested eggs release oncospheres that are able to penetrate the human intestinal wall. These oncospheres enter the portal venous system, which provides access to the liver, lungs, and various other organs [[14], [15], [16]]. Next, the oncospheres begin cyst development. Cysts are usually unilocular, and can range anywhere from 1 cm to 15 cm in diameter. In CE, cyst growth ranges from 1–2 mm to 10 mm per year. They also tend to affect the right lobe more frequently than the left lobe due to the nature of portal blood flow. The cysts are composed of two layers of membrane: an inner, nucleated, germinal membrane, and an outer, acellular, laminated layer. The immune system responds to the cyst by forming a calcified fibrous capsule around it, which is the layer that is most often visualized on imaging studies [15]. The cyst enlarges to form a combination of protoscolices and daughter cysts. The combination of many protoscolices and cystic fluid appears grain-like on ultrasound imaging, and is thus termed “hydatid sand.” Animals that consume organs infected with protoscolices will become definitive hosts, as the protoscolices attach firmly to the host's intestine, and then develop into an adult worm with a scolex (head), neck, and proglottids [15,17]. *E. granulosus* infections usually present as solitary cysts, and have single-organ involvement. In 10–15% of patients, there can be involvement of two organs depending on the specific geographic region and strain of parasite [15].

## 4 Description of the pathogen

The echinococcal cyst is a fluid-filled, spherical, unilocular cyst that consists of an inner germinal layer of cells supported by a characteristic acidophilic-staining, acellular, laminated membrane of variable thickness [18]. Each cyst is surrounded by a host-produced layer of granulomatous adventitial reaction. Small vesicles called brood capsules bud internally from the germinal layer and produce multiple protoscolices by asexual division. With time, internal septations and daughter cysts can form, disrupting the unilocular pattern typical of the young echinococcal cysts.

## 5 Clinical features

CE can go undetected for many years due to the slow growth and development of cysts and host's immune system [19,20]. Depending on the size and location, cysts can eventually exert pressure on nearby structures, producing abdominal discomfort and pain [13,15,16]. Symptoms develop slowly. Epigastric and/or right hypochondriac pain, nausea and vomiting are frequently observed. 85–90% of the cases have single organ involvement, and more than 70% of patients have a single cyst. Based on the organ in which the cyst settles and the environment they affect, they may show various clinical manifestations changing from cholangitis with biliary ruptures, portal hypertension, biliary obstruction and fistulae, and ascites to abscess formation [21]. For example, cysts in the liver can compress bile ducts, causing obstruction that can manifest as obstructive jaundice, abdominal pain, anorexia, and pruritus [22]. When in the lungs, cysts can irritate the membranes leading to chronic cough, dyspnea, pleuritic chest pain, and haemoptysis [15,23]. Cyst rupture or leakage can cause immunologic symptoms from elevated levels of immunoglobulins (Ig). IgE, IgG2, and IgG4 have been implicated for allergic reactions such as pruritus, hives, and anaphylactic shock [20]. Ruptured cysts can release viable cystic contents and protoscolices into the peritoneum, resulting in secondary hydatidosis [23]. Thus, infectious symptoms can manifest as sepsis, either due to the primary infection or to a secondary infection from leakage into the biliary tree. In one study, bacterial superinfection was found in 7.3% (37/503) of patients diagnosed with CE [25]. Four of these patients developed severe sepsis, out of which two patients died. Bacteria most commonly seen in the liver cyst infections includes *E. coli*, Enterococcus, and Streptococcus viridans.

Most primary infections in humans consist of a single cyst; however, 20–40% of individuals have multiple cysts or multiple organ involvement. Even though infections may be acquired in childhood, most cases of liver and lung cysts become symptomatic and are diagnosed in adult patients because of the slowly growing nature of the echinococcal cyst. Only 10–20% of cases are diagnosed in patients younger than 16 years. However, cysts located in the brain or an eye can cause clinical symptoms even when small; thus, most cases of intracerebral echinococcosis are diagnosed in children. In the lungs, ruptured cyst membranes can be evacuated entirely through the bronchi or can be retained to serve as a nidus for bacterial or fungal infection. Dissemination of protoscolices can result in multiple secondary echinococcosis disease. Larval growth in bones is atypical; when it occurs, invasion of marrow cavities and spongiosa is common and causes extensive erosion of the bone [6].

## 6 Complications

Cyst may rupture into the biliary system (leading to cholangitis with or without obstructive jaundice and marked eosinophilia), into the peritoneum (leading to anaphylaxis and/or peritoneal dissemination) or into the pleura or lung (causing pleural hydatidosis or bronchial fistula) [26]. The rupture may either be spontaneous or more usually after blunt trauma. Rupture of cyst can be of three types [27]:

1. Contained rupture - only endocyst is torn and cyst contents are confined within pericyst. The size of cyst does not decrease on imaging.
2. Communicating rupture - there is tearing of endocyst and cyst contents escape via biliary radicals or bronchioles that have been incorporated in pericyst. On imaging, cyst becomes smaller with undulating membrane.
3. Direct rupture - both endocyst and pericyst rupture causing spillage of contents into peritoneum or pleural space and dissemination of disease.

Cysts may become infected following bacteremia or via communicating bile ducts, especially when endoscopic retrograde cholangio-pancreatography (ERCP) has been performed. These patients present with high fever, sepsis syndrome and a tender liver. Pressure or mass effect on the bile ducts, portal veins, hepatic veins and inferior vena cava can cause cholestasis, portal hypertension and the Budd-Chiari Syndrome, respectively.

## 7 Diagnosis

The diagnosis is based on history, clinical examination, Serology and Imaging. Microscopic examination of the cyst content confirms the diagnosis. Diagnosis from serum studies is difficult because of the low sensitivity, which is frequently due to undetectable immune responses [28]. Immune responses depend on the location, cyst wall intactness, and viability of the organisms. Serum liver enzyme tests also have low sensitivities, and are frequently unreliable in determining the underlying severity of the infection. Moreover, liver enzyme tests are abnormal in only 40% of CE infected patients. When present, alkaline phosphatase is commonly elevated, while aspartate/alanine transaminase ratio and bilirubin levels typically remain within the normal limits. Complete blood count tests may be helpful, as Eosinophilia may be present in 40% of patients [23]. (see Flowchart 1 and Flowchart 2)

**Fig. 1:**
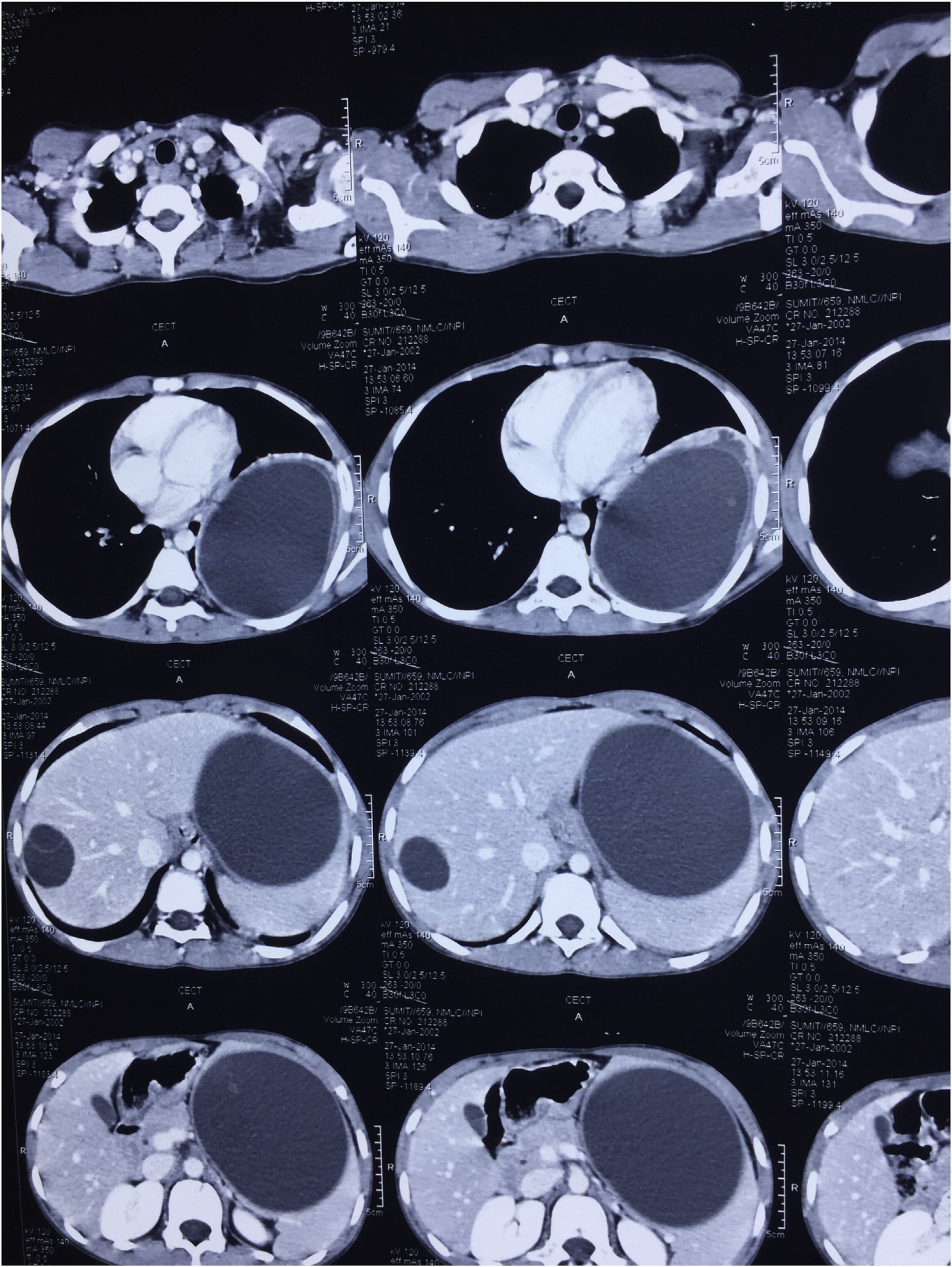
CT image after contrast enhancement showing hydatid cysts in liver, lung and spleen in different sections.

**Fig. 2:**
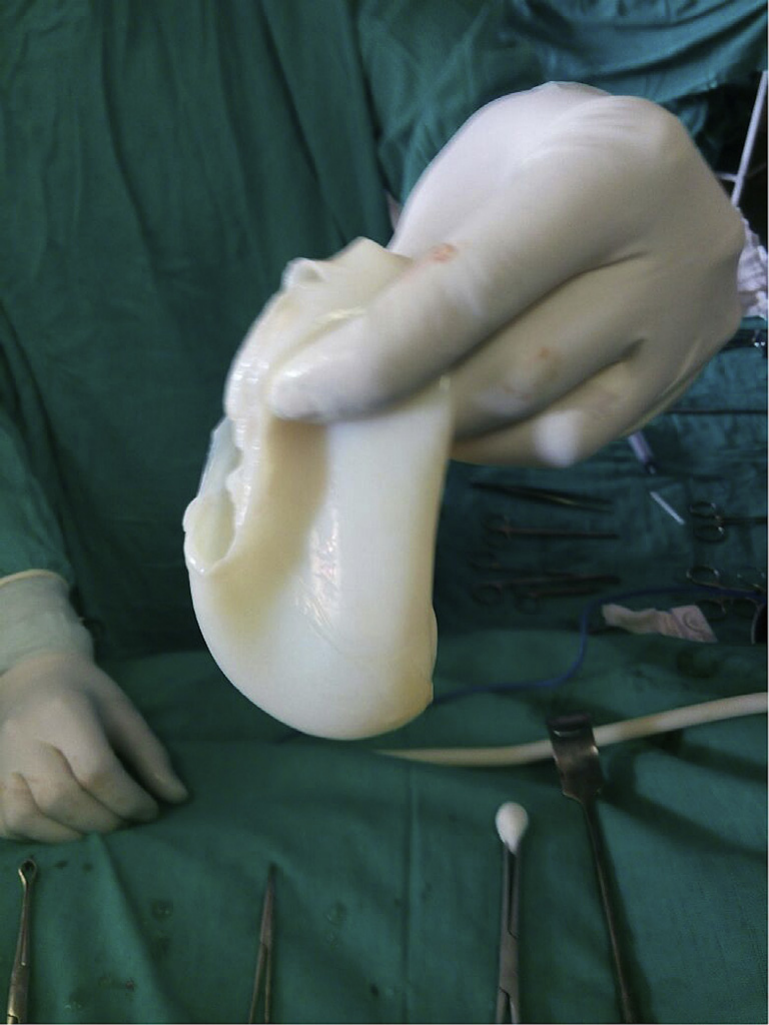
Pericystectomy specimen after draining the contents.

**Flowchart 1:**
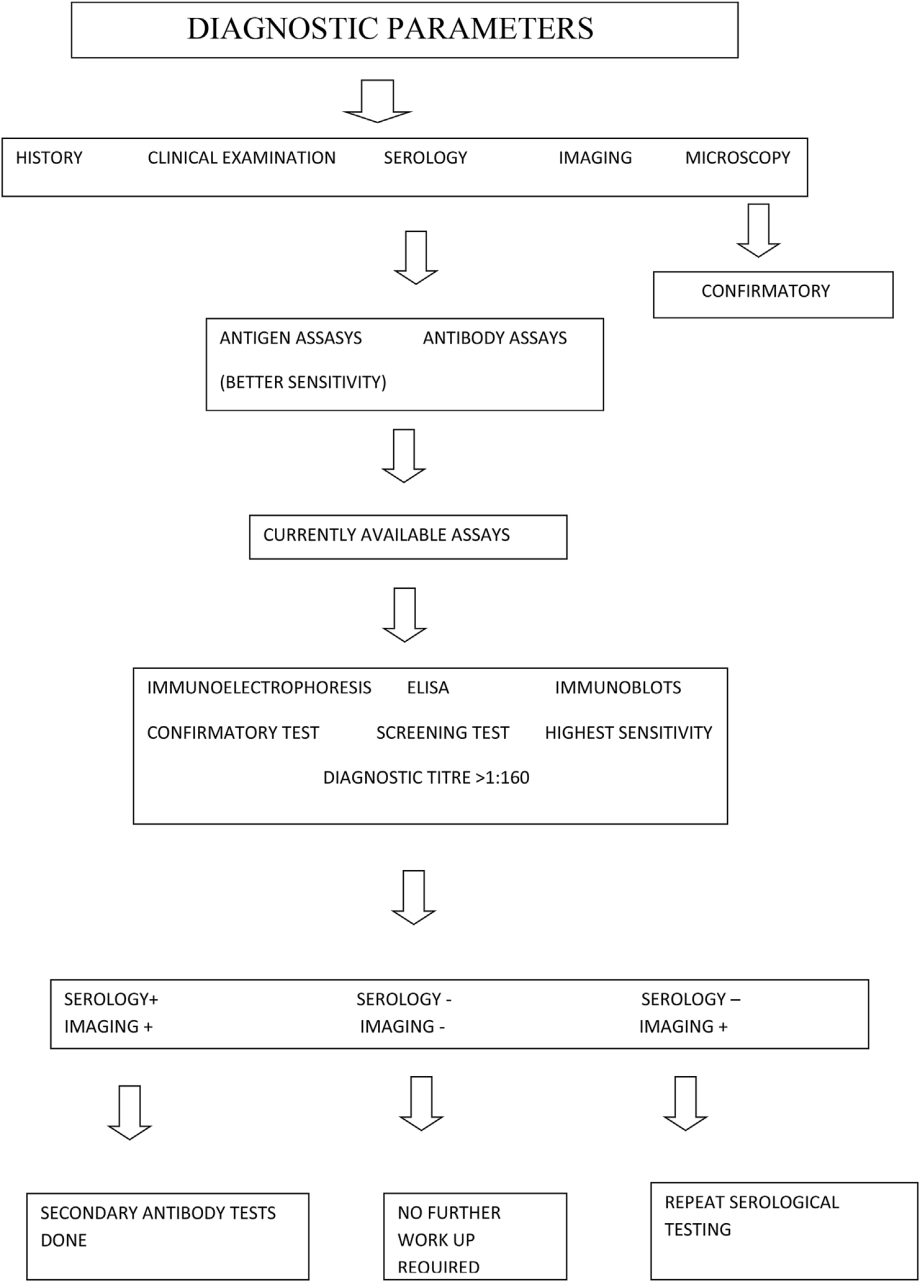
Diagnostic approach in a case of hydatid cyst.

**Flowchart 2:**
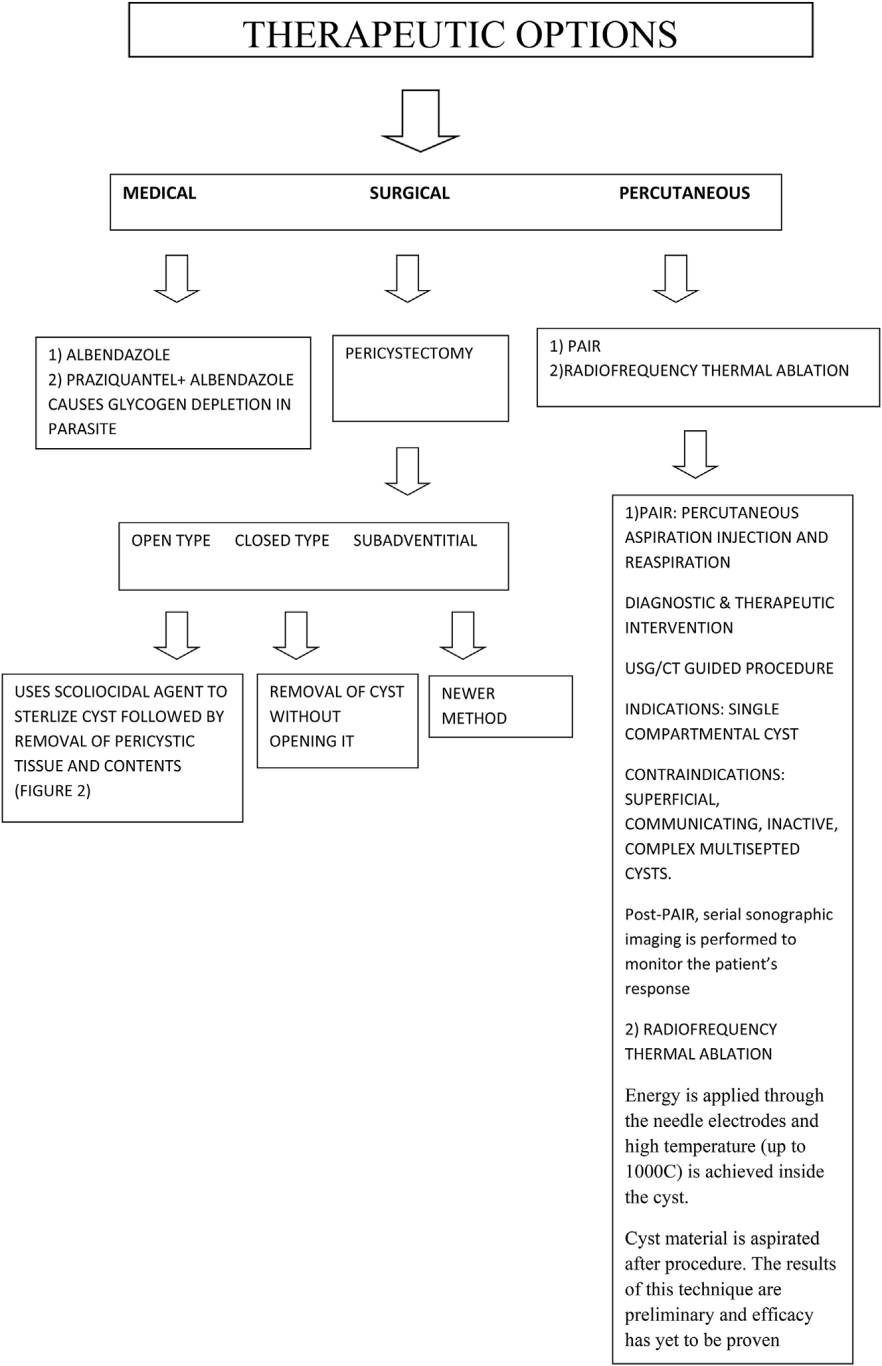
Therapeutic approach in a case of hydatid cyst.

## 8 Treatment

The approach to management and treatment of CE depends on the extent of organ involvement, the number of cysts, presence or absence of cystic–biliary communications and other factors, such as secondary bacterial infection and hemorrhage [23]. The ultimate goal of treatment is elimination of the germinal layer. Currently, three treatment options are available: Medical, Surgical and Percutaneous.

Medical therapy is indicated in the following cases: 1) in-operable cases in primary lung and liver CE with multiple cysts and peritoneal involvement; 2) to reduce cyst pressure, secondary seeding, and risk of recurrence in pre-surgical and pre-puncture cases. Contraindications include: 1) large cysts that are likely to rupture; 2) inactive or heavily calcified cysts; 3) early pregnancy; 4) chronic hepatic conditions and bone marrow suppressive disorders where treatment results in adverse side effects [10].

In general there are four different goals of medical treatment. (i) Definite cure – for univesicular cyst (type 1 and 2) 3–6 month treatment has success rate of 82% and relapse rate of above 25%, most of which occurs within 2 years. Life-long follow-up is advised. (ii) Reduction of viability and cyst size can be achieved in multi-vesicular cysts (type 3), however, germinal layer is rather resistant to treatment and definite cure occurs infrequently, (iii) Pre-operative reduction of viability of univesicular cysts before planning elective surgery or percutaneous drainage, (iv) Peri-operative or peri-interventional prophylaxis –optimally should be started at least 3 days before the surgical percutaneous treatment and should be continued for 3–8 weeks post-treatment in uncomplicated cases and for 3–6 months in complicated cases.

Surgical management, most commonly with partial and total cystectomy, has long been considered the definitive cure for CE [29]. The aim of surgery is total removal of the cyst with avoidance of the adverse consequences of spilling the contents. Approaches vary from radical resection to simple cyst resection, but each case varies depending on location, number of cysts, and structural complications, with the ideal approach being whole, simple resection without rupture [30].

## 9 Follow-up period

Follow-up is recommended initially every six months for the first two years, and then once a year depending on the appropriate clinical setting. In CE, it is difficult to assess the frequency of relapses. Therefore, monitoring with ultrasound is sometimes performed for up to ten years, a duration for which recurrences have been reported despite treatment. In the post-treatment phase, serologic studies, often with Ig levels, are difficult to interpret because they may indicate residual disease as opposed to a disease recurrence. In many cases, they remain elevated despite appropriate therapy or complete resection, which is why they are often used in combination with imaging studies during follow-up to detect cystic activity [8].

## 10 Prevention and control

The earliest successful control program was in Iceland initiated nearly 130 years ago, when hydatid disease was affecting approximately one in every six Icelanders. An extremely effective health education campaign sensitized the entire population, and subsequent measures virtually eliminated home slaughter of sheep resulting in the gradual elimination of transmission. By the 1950s, echinococcosis was considered eradicated from Iceland. Programs initiated in New Zealand (1959) and in Tasmania (1965) were primarily based upon education of rural populations and motivating them to change their practices. Strict control and prohibition of farm slaughter were key features in those programs. The initially voluntary nature of the programs was reinforced by legislative acts and strengthened efforts at enforcement as the programs progressed. Cystic echinococcosis has been declared provisionally eradicated in both Tasmania and New Zealand. Tasmania quarantined infected dogs and infected sheep flocks. Regional programs in Argentina (1970), Chile (1978), and Uruguay benefited from the use of the highly effective echinococcicidal drug praziquantel. Surveillance data from all these programs documented the reduction of prevalence in dogs, animal intermediate hosts, and humans [32]. A promising advance has been the development of a recombinant vaccine (EG95), which seems to confer 96–98% protection against infection. Recent trials in Australia and Argentina using EG95 have reported that 86% of vaccinated sheep were completely free of viable hydatid cysts when examined 1 year after immunization. Vaccination reduced the number of viable cysts by 99.3% [33]. A vaccine has also been developed against the dog tapeworm stage, which conferred 97–100% protection against worm growth and egg production [34]. It must be noted that the positive achievements of successful control programs, however significant at the local level, have not markedly changed the global distribution and public health importance of hydatid disease. In most endemic areas, effective control has not been achieved or even attempted. Much remains to be done. There is concern that echinococcosis may have become hyperendemic in areas where it was once endemic.

## 11 Conclusion

Echinococcal cysts, although fairly uncommon, should be considered in the differential diagnosis of hepatic cysts, particularly in patients with exposure risk. Imaging is crucial in determining cyst stage, size, location and complications. It can also be helpful in assessing the suitability of a minimally invasive PAIR approach. Uncomplicated active cysts can be managed with chemotherapy alone or in combination with a PAIR approach. Uncomplicated, inactive cysts can be managed with the “watch-and-wait” strategy. Surgery is the first choice when there is cysto-biliary fistula, significant extra-hepatic extension with high risk of perforation, complicated cysts (ruptured or infected) and when expertise to percutaneous treatment is not available. Hydatid cyst should be considered in the differential diagnosis of the following pathologies: Simple cysts, non-organized haematoma, necrotic tumor, cystic metastatic carcinoma, haemangioma, pyogenic abscesses, amoebic abscess, tuberculosis and fungal infections.

## Conflicts of interest

None.

## Source of funding

None.

## Ethical approval

Not applied as it is a review article.

## Research registration number (UIN)

Not applicable.

## Trial registry number

NA.

## Author contribution

Namita Bhutani: Study design, data collection and wrote the article.

Pradeep Kajal: Final editing and contributed the clinical part.

## Guarantor

Pradeep Kajal.

## Provenance and peer review

Not commissioned, peer reviewed.
